# Structural Mimicry of Receptor Interaction by Antagonistic Interleukin-6 (IL-6) Antibodies*[Fn FN2]

**DOI:** 10.1074/jbc.M115.695528

**Published:** 2016-04-27

**Authors:** Christophe Blanchetot, Natalie De Jonge, Aline Desmyter, Nico Ongenae, Erik Hofman, Alex Klarenbeek, Ava Sadi, Anna Hultberg, Anke Kretz-Rommel, Silvia Spinelli, Remy Loris, Christian Cambillau, Hans de Haard

**Affiliations:** From ‡ argenx, 9052 Zwijnaarde, Belgium,; § Architecture et Fonction des Macromolécules Biologiques, UMR 6098 CNRS and Universités of Marseille, 13284 Marseille, France,; ¶ Bird Rock Bio, La Jolla, California 92037,; ‖ Structural Biology Brussels, Vrije Universiteit Brussel, 1050 Brussels, Belgium, and; the ** Structural Biology Research Center, Instituut voor Biotechnologie, 1050 Brussels, Belgium

**Keywords:** antibody engineering, crystal structure, immunology, interleukin 6 (IL-6), structure-function, high affinity

## Abstract

Interleukin 6 plays a key role in mediating inflammatory reactions in autoimmune diseases and cancer, where it is also involved in metastasis and tissue invasion. Neutralizing antibodies against IL-6 and its receptor have been approved for therapeutic intervention or are in advanced stages of clinical development. Here we describe the crystal structures of the complexes of IL-6 with two Fabs derived from conventional camelid antibodies that antagonize the interaction between the cytokine and its receptor. The x-ray structures of these complexes provide insights into the mechanism of neutralization by the two antibodies and explain the very high potency of one of the antibodies. It effectively competes for binding to the cytokine with IL-6 receptor (IL-6R) by using side chains of two CDR residues filling the site I cavities of IL-6, thus mimicking the interactions of Phe^229^ and Phe^279^ of IL-6R. In the first antibody, a HCDR3 tryptophan binds similarly to hot spot residue Phe^279^. Mutation of this HCDR3 Trp residue into any other residue except Tyr or Phe significantly weakens binding of the antibody to IL-6, as was also observed for IL-6R mutants of Phe^279^. In the second antibody, the side chain of HCDR3 valine ties into site I like IL-6R Phe^279^, whereas a LCDR1 tyrosine side chain occupies a second cavity within site I and mimics the interactions of IL-6R Phe^229^.

## Introduction

IL-6 is a cytokine that was discovered in the 1980s and is produced by several types of cells from the innate immune system, as well as by other cells including those from several types of cancer (1). IL-6 is activated by tissue damage and stress, and elevated IL-6 concentrations contribute to several inflammatory diseases and malignancies. In addition to its role in inflammation, IL-6 is involved in numerous cellular processes and associated diseases including cardial infarction, renal disease, cognitive dysfunction, atherosclerosis, and cancer, to name just a few. It is therefore no surprise that IL-6 and its receptor should be actively investigated as potential drug targets in a number of contexts.

IL-6 signaling is transmitted through gp130 (2), a transmembrane protein ubiquitously expressed and associated with a variety of cytokine receptors to activate the Janus family of tyrosine kinases (JAK), in particular JAK1, JAK2, and Tyk2 (3, 4). Communication between IL-6 and gp130 occurs through the IL-6 receptor (IL-6R, also called gp80) (5).^3^ The extracellular domains of IL-6R and gp130 associate with IL-6 to form a ring-shaped hexameric complex with 2:2:2 stoichiometry. Although gp130 is present on a plethora of cell types (6), IL-6R expression is limited to innate immune cells. Cells that do not express IL-6R can still be regulated by IL-6 through a so-called “trans signaling” mechanism (7, 8). The extracellular part of the receptor is shed from the surface of IL-6R presenting cells (mostly neutrophils and macrophages) by a proteolytic cleavage that produces soluble IL-6R (9–11). This allows IL-6 to have effects on non-leukocyte cells that express gp130, such as cancer cells, fibroblasts, and epithelial cells (12–14).

Antibodies that block the interaction between IL-6 and its receptor have been successfully used in treatment for inflammatory diseases (12, 15–17). Tocilizumab, a humanized anti-IL-6R antibody that binds both cell surfaces of IL-6R and soluble IL-6R, is approved for treatment of rheumatoid arthritis as well as systemic juvenile idiopathic arthritis and shows great promise against Crohn disease (18, 19). Other antibody-based therapies target IL-6 itself with a view to treat rheumatoid arthritis, metastatic castration-resistant prostate cancer, and nonmalignant gastrointestinal diseases (20). Of these, the structure of a complex between IL-6 and the Fab fragment has been obtained only for olokizumab (21). Here we present the crystal structures of IL-6 in complex with two novel IL-6-neutralizing camelid Fab fragments, 61H7 and 68F2 that mimic the interaction of IL-6 with IL-6R.

## Experimental Procedures

### 

#### 

##### Generation of Llama-derived Antibodies 61H7 and 68F2

Immunization with recombinant IL-6, phage Fab library construction, selection, screenings, chain shuffling, production, and purification of antibodies have been previously described (22).

##### Protein Production

8 mg of antibodies 61H7 and 68F2 (4 mg/ml) in Dulbecco's PBS (d-PBS), pH 7.2, were exchanged to digestion buffer containing 20 mm cysteine HCl on a Zeba^TM^ desalt spin column (Fab preparation kit; Pierce Thermo Scientific). Samples were incubated with immobilized papain (Pierce Thermo Scientific) and digested for 6 h at 37 °C. The Fc fragments were separated from the Fab fragments using a CaptureSelect human Fc affinity matrix (BAC BV Unilever) equilibrated in d-PBS. Fab fragments were recovered in the flow-through, and Fc fragments were eluted using 0.1 m glycine, pH 2.0. Protein concentration was determined by UV spectrometry from the absorbance at 280 nm. 4.6 mg (>50%) of purified Fab was recovered and concentrated to 1.25 and 1.53 mg/ml, respectively, on Amicon-Ultra filters (cutoff, 10 kDa).

##### IL-6·61H7 or 68F2 Fab Complex Purification

2.5 mg of recombinant human IL-6 (Immunotools) was incubated with 2.6 mg of Fab from 61H7 or 68F2 in Dulbecco's Phosphate buffered saline (d-PBS) pH 7.2 for 1 h at 4 °C before being concentrated to 1 ml on a Amicon-Ultra filter (cutoff, 10 kDa). The IL-6·61H7 or 68F2 complexes were then separated from excess free IL-6 by size exclusion chromatography on a Superdex 75 column in d-PBS and finally concentrated to respectively 8.45 and 8.1 mg/ml on an Amicon-Ultra concentrator (cutoff, 10 kDa). Purification of the complexes was evaluated with SDS-PAGE.

Quality control on the purified complexes was performed by size exclusion chromatography on an Alliance 2695 HPLC system (Waters) using a Silica Gel KW803 column (Shodex) eluted with 50 mm Tris-HCl, pH 7.5, 150 mm NaCl at a flow rate of 0.5 ml/min. Detection was performed using a triple-angle light scattering detector (Mini-Dawn^TM^ TREOS, Wyatt Technology, Santa Barbara, CA). Molecular weight determination was performed by ASTRA V software (Wyatt Technology).

##### Crystallization and Data Collection

Initial crystallization screening of the IL-6·61H7 and 68F2 Fab complexes was performed with commercial kits structure screen 1 and 2, Proplex screen and Stura Footprint screen (Molecular Dimensions Ltd.). Drops were set up with a 1:1 (v/v) ratio of protein (at 8.45 or 8.1 mg/ml, respectively) to mother liquor in a total volume of 200 nl on Greiner 96-well plates using a Cartesian MicroSys SQ robot (23).

Diffraction quality crystals of complex IL-6·61H7 were obtained by sitting drop vapor diffusion at 277 K after optimization in 27.14% PEG MME 2K, 0.1 m Na-Hepes, pH 7.14. Crystal for data collection was transferred to mother liquor with 7.5% ethylene glycol and flash-frozen in liquid nitrogen. Diffraction data were collected on Beamline Proxima1 (Soleil). The crystals belong to the C2 space group with unit cell dimensions: *a* = 108.2 Å, *b* = 47.5 Å, *c* = 148.3 Å, and β = 97°. Diffraction data were collected up to 2.9 Å resolution at 100 K at Soleil (Saint-Aubin, France) at a resolution of 2.3 Å. The crystals of the IL-6·61H7 complex contain one IL-6·Fab complex per asymmetric unit with a V*_m_* value of 2.36 Å^3^/Da, which corresponds to a solvent content of 48%.

A diffraction quality crystal of complex IL-6·68F2 was obtained by sitting drop vapor diffusion at 277 K after 9 months in 25% PEG 4K, 0.15 m (NH_4_)_2_SO_4_, 0.1 m MES, pH 5.5. Crystal for data collection was transferred to mother liquor with 10% ethylene glycol and flash-frozen in liquid nitrogen. Diffraction data were collected up to 2.9 Å resolution at 100 K on Beamline ID14-4 at the European Synchrotron Research Facilities Synchrotron (Grenoble, France), using an ADSC Quantum 4 detector. Data were processed with XDS and scaled with XSCALE (24). The crystal structure of IL-6 in complex with Fabs 61H7 or 68F2 was determined by molecular replacement with Fab structures and the IL-6 structure using MolRep (25). Refinement of the complexes was performed with auto BUSTER (26). Data collections and refinement statistics are presented in Table 1. The data have been deposited with the Protein Data Bank under the accession codes 4O9H (IL-6 in complex with Fab 61H7) and 4ZS7 (IL-6 in complex with Fab 68F2).

**TABLE 1:** **Proliferation assay using the B9 or 7TD1 cell line in presence of 61H7 or 68F2 dilutions to neutralize the effect of human IL-6 (IC_50_ pm)** The affinities (*K_D_* pm) of antibodies 61H7 and 68F2 were measured by SPR.

mAb	Proliferation assay (IC_50_ pm)		Affinity SPR (*K_D_* pm)*^a^*
	B9	7TD1	
61H7	1.7	3.5	3.3–6.3
68F2	0.6	0.7	13–21

*^a^* Dissociation kinetics outside the detection limit of the Biacore T200.

##### Competition Assays

Competition ELISA was performed as follows: non-neutralizing IL-6R antibody (BN12; Diaclone) was immobilized on Maxisorp plate at a concentration of 1 μg/ml overnight at 4 °C. Then 0.1 μg/ml of IL-6R (R&D Systems) was incubated 1 h at room temperature. Biotinylated human IL-6 (0.025 μg/ml) alone or in combination with a concentration series of antibody was added for 1 h at room temperature. After washing, biot-IL-6 bound to the IL-6R was detected with Strep-HRP. After addition of TMB and H_2_SO_4_ to stop the reaction, optical density was read at 450 nm. IL-6 was biotinylated using the Pierce kit with the modification that the biotinylation reaction was performed at pH 5.5 to only biotinylate the N terminus of IL-6. Antibody competition with biot-IL-6 for IL-6R binding was expressed as a percentage of biot-IL-6 binding as compared with biot-IL-6 alone using GraphPad Prism v6.

Surface plasmon resonance (SPR; Biacore 3000) was used for competition experiments on a low density IL-6 coating (∼75–100 resonance units). mAb 61H7 was first injected at 50 μg/ml with a flow rate of 30 μl/min. A second antibody (50 μg/ml of 61H7 or 68F2) was added using COINJECT procedure at the same flow rate to investigate competition for binding to coupled IL-6.

##### HCDR3 Mutagenesis and Screening

Mutations in HCDR3 were generated by overlap extension PCR using the plasmid pCB4-111A7 (containing the variable domains of 61H7 with few mutations in the framework to improve human identity without affecting affinity and fused to the human constant domain CH1 and Cλ) as template (50 ng) and Phusion^TM^ DNA polymerase (Thermo Scientific). Briefly, the DNA fragment containing frameworks 1–3 was generated using primers PelB3 (GCGCCAATTCTATTTCAAGG) and VH_W98X (5′ACCTGCACGATTTGCACAATAATAAACTGCGGTG 3′). The DNA fragment containing CDR3-FR4-CH1 product was generated with two different degenerated sense primers, one with a leucine at position 100 (VH_W98XL100, 5′-GTGCAAATCGTGCAGGT*NNK*GGTCTGGGTGATTATTGGGGACAGGGG-3′) and one with alanine at position 100 (VH_W98XA100, 5′-GTGCAAATCGTGCAGGT*NNK*GGTGCGGGTGATTATTGGGGACAGGGG-3′) and M13 (GCCAGGGTTTTCCCAGTCACGA) as antisense primer. The products were purified separately and combined for the final overlapping PCR using primers PelB3 and M13. The PCR amplicons were gel-extracted and cloned back into pCB4 Fab expression vector (phagemid devoid of pIII) containing the light chain of 111A7 and finally transformed into TG1 *Escherichia coli* cells. After HCDR3 mutagenesis, single bacterial clones were grown at 37 °C (while shaking at 180 rpm) in 2TY medium containing 100 μg/ml ampicillin in 96 deep well plates (Nunc). When optical density at 600 nm reached between 0.8 and 1.0, isopropyl β-d-1-thiogalactopyranoside was added to a final concentration of 1 mm to induce Fab expression in the bacterial periplasm. After overnight growth of the cells at 28 °C, periplasmic contents were extracted by a cycle of freezing (overnight, at −20 °C) and thawing in 100 μl of PBS (at 4 °C) causing cell lysis. After 1 h shaking at room temperature, the cells were pelleted again, and the PBS supernatant containing the periplasmic extract (containing the mutant Fabs) was used to determine the off rate (*k*_off_) by SPR, whereas the clones are sequenced.

##### Surface Plasmon Resonance

SPR was performed using a Biacore 3000 and low density IL-6 coating (∼75–100 resonance units) on a CM5-Chip (GE Healthcare) to determine the *k*_off_ of Fab mutants produced in periplasmic fractions as previously described (22). *k*_off_ was measured over a 10-min washing period (30 μl/min) and evaluated using the BIAevaluation software. Affinity measurements of mAbs 61H7 and 68F2 were performed using a Biacore T200 and are described in supplemental Fig. S1.

##### Proliferation Assay Using IL-6-dependent 7TD1 and B9 Cell Lines

The proliferation assay using 7TD1 or B9 cells was performed as previously described (22, 27, 28).

## Results

### 

#### 

##### Overall Structures of the IL-6·Fab Complexes

From the amplified antibody repertoire of two llamas immunized with human IL-6, two leads with differing HCDR3 sequences were selected and their affinities further optimized by heavy chain and light chain shuffling. The two resulting mAbs and their Fabs will be further referred to as 61H7 and 68F2. For 61H7, heavy chain shuffling delivered a variant VH with identical HCDR3 sequence, hence derived from a common B cell clone, but with somatic mutations in HCDR1 and HCDR2, resulting in a potency of 3.5 and 1.7 pm as measured by blockade of IL-6-driven proliferation of 7TD1 and B9 cells, respectively (Table 1). Light chain shuffling of 68F2 yielded a variant λ light chain with identical LCDR3 sequence but with mutations in LCDR1 and LCDR2; the lead reformatted into human IgG1 turned out to have a potency as high as 60 and 70 fm as measured in the B9 and 7TD1 cell line based assays, respectively (Table 1). The affinity (*K_D_*) to IL-6 was estimated to be 63 pm for 61H7 and 21 pm for 68F2 as measured with SPR, although this could be underestimated because of the sensitivity limit of the machine (Table 1 and supplemental Fig. S1).

**FIGURE 1.**
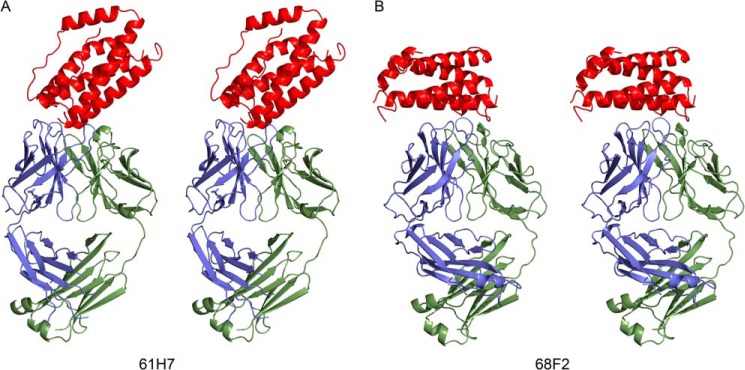
**Structure of the Fab complexes with IL-6.** *A*, stereo view of 61H7 bound to IL-6. *B*, stereo view of 68F2 bound to IL-6. In each panel the cytokine is shown in *red*, the VH and CH1 domains of the Fabs are in *blue*, and the VL and CL domains are in *green*.

To better understand the very high affinity of the two antibodies the crystal structures of human IL-6 in complex with 61H7 and 68F2 were determined at 2.4 and 2.9 Å resolution, respectively. Both structures were refined to low *R* values and good stereochemistry. In each case, the asymmetric unit contains a single Fab·IL-6 complex with 1:1 stoichiometry (Table 2 and Fig. 1). In both cases, a large interaction surface is observed involving both VH and VL chains. For the complex of IL-6 with 61H7, 940 Å^2^ of the Fab is buried, 60% of which concerns VH and 40% VL. The interactions are limited to the CDR1 and CDR3 of the light chain and the CDR1, CDR2, and CDR3 of the heavy chain. In the complex with 68F2, VH (50%) and VL (50%) contribute equally to the large interaction surface (1156 Å^2^). Analogous to the 61H7·IL-6 complex, only the light chain CDR2 loop is not directly involved in the interaction. The full set of interactions (hydrogen bonds and salt bridges) observed between antibody and antigen are listed in supplemental Tables S1 and S2.

**TABLE 2:** **Crystallographic data collection and refinement statistics of structures of complexes of IL-6 with 61H7 and 68F2**

	IL-6–61H7	IL-6–68F2
**Data collection**		
Protein Data Bank code	4O9H	4ZS7
Beamline	Proxima 1 (SOLEIL)	European Synchrotron Research Facilities Synchrotron ID14-4
Space group/cell dimensions	C2/*a* = 108 2 Å, *b* = 47.5 Å, *c* = 148.3 Å, β = 97°	P2/*a* = 46.5 Å, *b* = 92.9 Å, *c* = 84.6 Å, β = 103.9°
Resolution limits (Å)*^a^*	45.0–2.36 (2.42–2.36)	45.0–2.93 (3.0–2.93)
*R*_merge_ (%)*^a^*	16.0 (65.0)	18.0 (87)
No. of observations*^a^*	116,261 (14,546)	95,495 (5067)
No. unique reflections*^a^*	31,185 (2201)	14,778 (969)
Mean ((*I*)/S.D. (*I*))*^a^*	14.2 (1.8)	7.5 (2.3)
Completeness (%)*^a^*	99.4 (98.1)	97.9 (88.8)
Multiplicity*^a^*	3.6 (3.5)	6.5 (5.2)

**Refinement**		
Resolution (Å)*^a^*	38.6–2.36 (2.44–2.36)	44.3–2.93 (3.17–2.93)
No. of reflections*^a^*	31,036 (2635)	14,778 (2604)
No. of protein/water atoms	4306/235	4374/80
No. test set reflections	1552	1490
*R*_work_/*R*_free_ (%)*^a^*	22.5/25.9 (26.7/33.5)	26.7/29.6 (28.9/34.4)
RMSD bonds (Å)/angles (°)	0.010/1.38	0.009/1.39
*B*_Wilson_/*B*_average_	52.4/67.7	45.6
Coot's Ramachandran (preferred/allowed/outliers, %)	92/6/2	88.4/ 8/3.6

*^a^* Parentheses refer to the highest resolution bin.

Compared with olokizumab, which binds at a distinct epitope (21), for all three antibodies the heavy chain contributes approximately the same amount of binding surface (∼590 Å^2^ for 68F2 and 61H7, 520 Å^2^ for olokizumab) but differ mostly in their amount of light chain binding surface (580 Å^2^ for 68F2 compared with 320 Å^2^ for olokizumab). Olokizumab also shows a significantly larger portion of hydrophobic contact surface (56%) compared with 68F2 and 61H7 (38 and 40%, respectively) and even IL-6R (46%).

##### IL-6 Conformation

**FIGURE 2.**
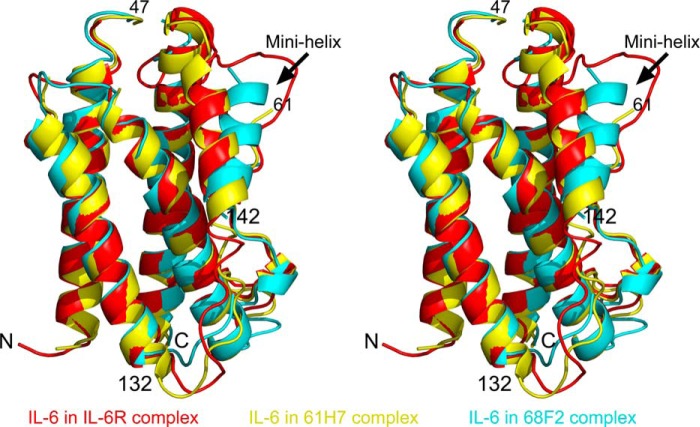
**Superposition of the IL-6 structure as found in the Fab cytokine complexes with published structures.** IL-6 from the IL-6·IL-6R complex (Protein Data Bank code 1P9M) is shown in *red*, IL-6 as present in the complex with 61H7 is in *yellow*, and the cytokine from its complex with 68F2 is in *cyan*. The difference in position of the mini helix as found in 68F2·IL-6 complex is indicated with an *arrow*.

The structures of IL-6 as present in both Fab complexes were compared with the structures of IL-6 in its free state and bound to IL-6R and gp130 (29, 30). Fab binding does not disturb the four-helix bundle motif that is the core of the IL-6 fold (Fig. 2). Superposition of apo IL-6 (Protein Data Bank code 1ALU) with the cytokine from the IL-6·61H7 complex shows a very close agreement (RMSD 0.54 Å for all Cα atoms). This conformation is distinct from the one observed in the receptor complex (Protein Data Bank code 1P9M), where the RMSD is increased to 1.2 Å. Two loops differ in conformation. The first one, covering residues Asn^48^–Asn^61^, is a long loop that is unstructured in the apo IL-6 and in the IL-6·61H7 complex. This loop is stabilized in the IL-6·IL-6R structure by the binding of IL-6R. The second loop that differs in conformation is the loop that connects the mini helix to the helix α3 (Asn^132^–Pro^141^).

The conformation of IL-6 in complex with 68F2 on the other hand is more deviant, with RMSD values ranging from 1.2 Å (IL-6 in the 61H7 complex) to >1.4 Å (apo IL-6) to >2.0 Å (complex with IL-6 and gp130). These differences result from a different orientation of residues 140–152, corresponding to the mini helix (Fig. 2). The latter rotates by roughly 25° around its N terminus. This movement is, however, not likely caused by the interaction with 68F2 because it is located on the opposite side of IL-6. Rather, because both loops on the N- and C-terminal sides of the mini helix lack density, the rearrangement may be due to loop cleavage.

##### Sterical Hindrance of IL-6R Interaction, but Not of gp130 Binding

**FIGURE 3.**
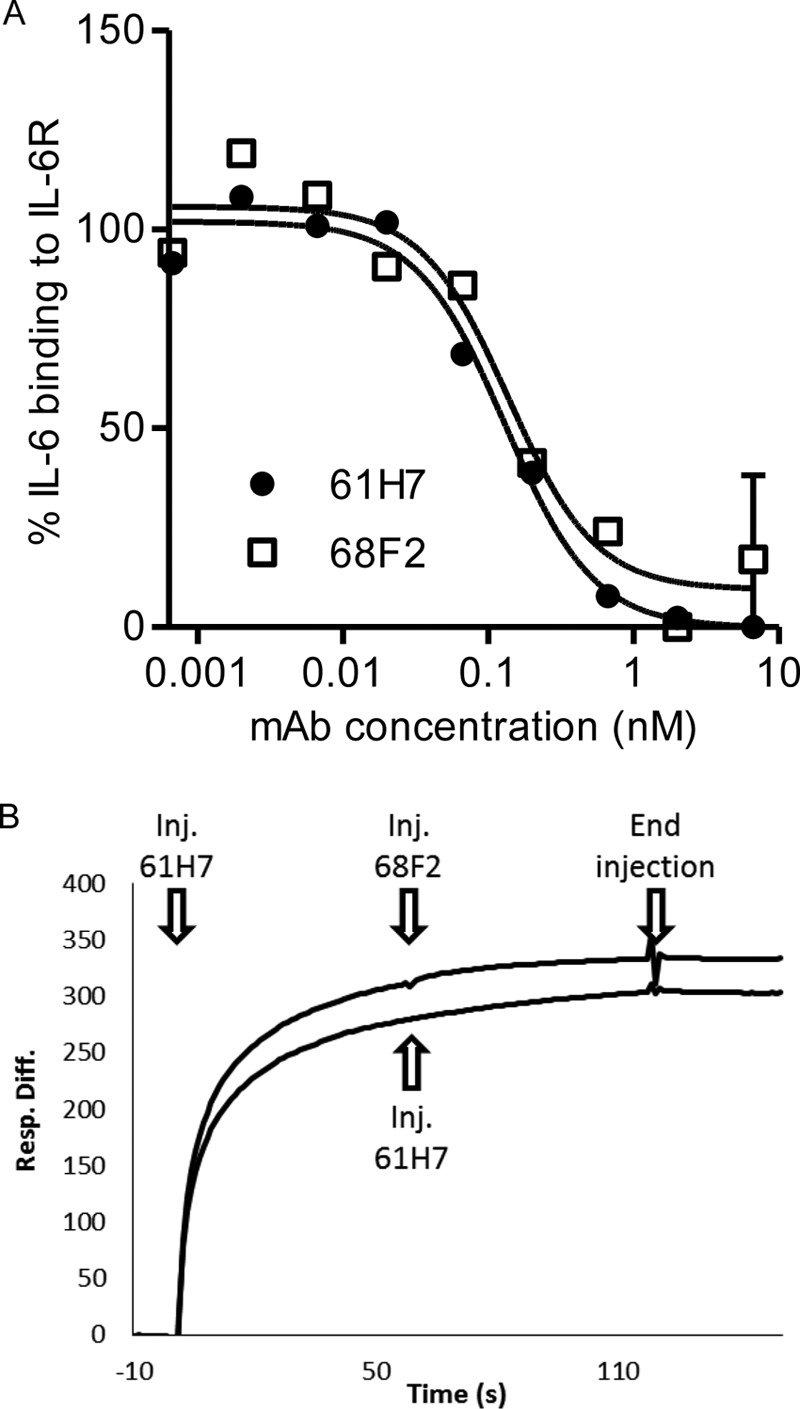
**Epitope mapping of the anti-IL-6 antibodies.** *A*, the ability of increasing concentrations of antibodies 61H7 and 68F2 to compete with biotinylated IL-6 for binding to captured IL-6R was investigated using a competition-based ELISA. IL-6 binding was revealed using horseradish peroxidase-conjugated streptavidin and expressed as a percentage of IL-6 binding to IL6-R as compared with IL-6 alone (*y* axis). The *x* axis shows mAb concentrations (nm). *B*, antibodies 61H7 and 68F2 compete with each other for binding to immobilized IL-6 as demonstrated by SPR. 61H7 (50 μg/ml) was first allowed to bind to and nearly saturate IL-6 (injected 61H7). Then 61H7 (injected 61H7, *lower curve*) or 68F2 (injected 68F2, *upper curve*) was added, and no additional binding was observed, indicating recognition of similar binding sites on IL-6. *Inj.*, injected; *Resp. Diff.*, response difference.

**FIGURE 4.**
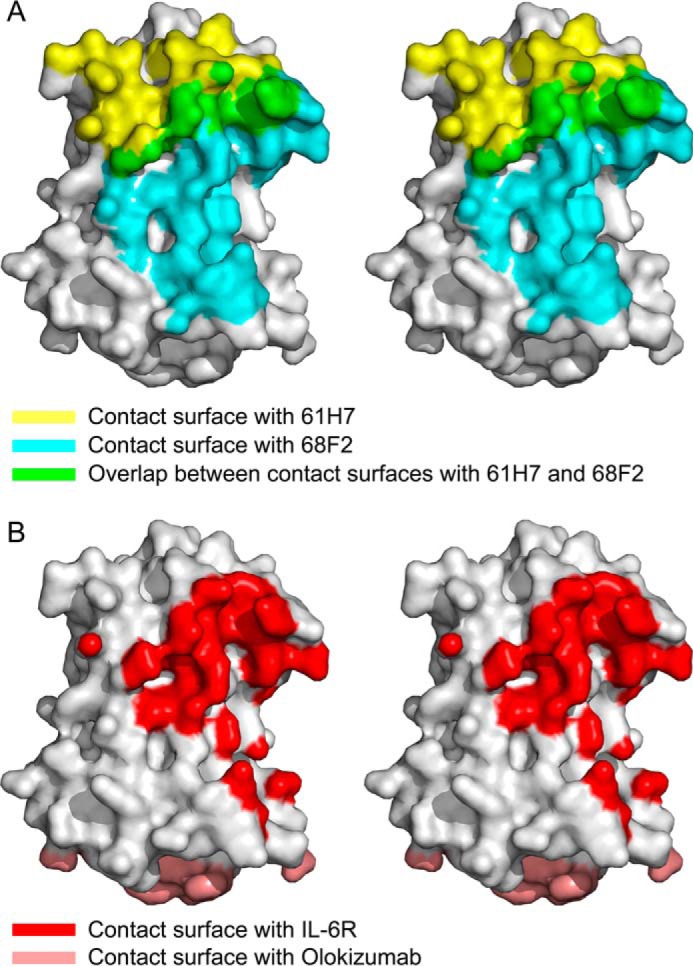
**Fab epitopes mapped on the surface of IL-6.** *A*, stereo view of a surface representation of IL-6 (free form; Protein Data Bank code 1ALU) with the surface covered by 61H7 colored *yellow*, and the surface covered by 68F2 is colored *cyan*. The overlap between both epitopes is colored *green. B*, identical representation of IL-6, but with the surface covered by IL-6R colored *red* and largely overlapping the one for 68F2. The epitope covered by olokizumab is shown in *pink* and located on the opposite side of IL-6.

In the ternary complex between IL-6, IL-6R, and gp130 (Protein Data Bank code 1P9M), there are three interaction interfaces involving IL-6 (30). They are termed sites I, II, and III, where site I corresponds to the interface between IL-6 and the D2 and D3 domains of IL-6R. It is worthwhile to mention that our antibodies were screened for their capability to block the interaction between IL-6 and IL-6R in an ELISA-based assay. Competition ELISA data show that both antibodies compete with IL-6 for IL-6R binding (Fig. 3*A*) and in an SPR-based assay with each other (Fig. 3*B*). Indeed, upon superposition of the respective IL-6 moieties, part of the VH domain of 61H7 overlaps with part of the VL domain of 68F2. Nevertheless, both antibodies recognize distinct yet partially overlapping epitopes. The overlap between both epitopes is rather small and is mainly formed by the VH paratope of 61H7 (Fig. 4).

68F2 binds on the side of IL-6, contacting the N-terminal part of α-helix 1 and the C-terminal part of α-helix 4 of the four-helix bundle, as well as the long loop between α-helices 1 and 2. This binding interface of 68F2 to a major extent overlaps with site I recognized by IL-6R but is significantly larger by 380 Å^2^. This is for the most part due to a larger number of polar contacts (280 Å^2^) and only to a lesser extent (100 Å^2^) due to additional hydrophobic contacts. Indeed, although the IL-6·IL-6R interface only hosts 20 hydrogen bonds and 2 salt bridges, on the IL-6·68F2 interface, 20 hydrogen bonds and 7 salt bridges are found. The interaction also involves a significantly larger number of contacts involving the main chain of IL-6. There is otherwise no steric conflict between the bound 68F2 and gp130 (data not shown).

61H7 on the other hand binds on the top of the four-helix bundle near the N and C termini. Here the VL domain sterically interferes with the C-terminal domain of IL-6R (residues 195–295). However, the overlap of the 61H7 epitope and the site I surface concentrates around a cavity occupied by both the HCDR3 loop of 61H7 and the IL-6R molecule (see below). Again, the interface is more hydrophilic than observed for the IL-6·IL-6R complex and hosts 21 hydrogen bonds. A smaller number of steric conflicts are also present between the C-terminal domain of gp130 (Ser^257^–Thr^258^) and the VL domain of 61H7. It is not clear whether they can be relieved by changes in the corresponding loop conformation of gp130.

##### Structural Mimicry of IL-6R Binding by 68F2

**FIGURE 5.**
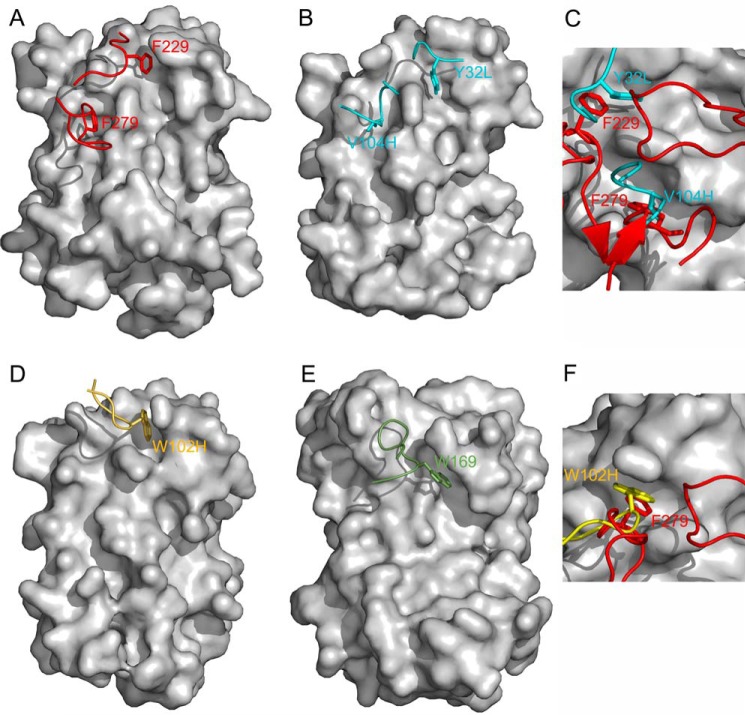
**Mimicry of site 1 interactions.** *A*, surface representation of IL-6 from the IL-6·IL-6R complex. The backbone trace of IL-6R residues 226–232 and 275–285 is shown in *red*. The side chains of Phe^229^ and Phe^279^ of IL-6R are shown in stick to illustrate their penetration into cavities on the site I surface of IL-6. *B*, equivalent surface representation, but for IL-6 in the IL-6·68F2 complex. Residues 31–35 of VL and 101–108 of VH of 68F2 are shown in *cyan*. Tyr^32^ of VL (indicated by *Y32L*) (Kabat numbering 30) and Val^104^ of VH (indicated by *V104H*) (*yellow*; Kabat numbering 99) mimic the interactions of Phe^229^ and Phe^279^ from IL-6R, respectively. *C*, superposition of key residues of 68F2 (in *cyan*) and IL-6R (in *red*) when bound to IL-6. *D*, surface representation of IL-6 from the IL-6·61H7 complex. The backbone trace of 61H7 residues 100–106 of VH is shown in *yellow*. Here the side chain of Trp^102^ of VH (indicated by *W102H*) (Kabat numbering 98) mimics Phe^229^ from IL-6R. *E*, surface representation of human growth hormone shown in the same orientation as IL-6 in *A*, *B*, and *D*. The backbone of residues 163–171 of the human growth hormone receptor is shown in *green*. Trp^169^ from the human growth hormone receptor penetrates into a cavity on the surface of human growth hormone that is located similarly as the site I cavity of IL-6, into which Phe^229^ of IL-6R penetrates. *F*, superposition of key residues of 68F2 (in *yellow*) and IL-6R (in *red*) when bound to IL-6.

Within site I, there are two small cavities on the surface of IL-6 that each accommodate a phenylalanine side chain from the D3 domain of IL-6R: Phe^229^ and Phe^279^ (Fig. 5*A*). Both contribute a significant fraction of contact area. Phe^229^ of IL-6R is called the “hot spot residue” by Boulanger *et al.* (30) because mutagenesis studies confirmed its critical role in the interaction between the receptor and the cytokine. Mutation of this residue to valine or serine completely abolishes the IL-6R binding to IL-6 (31).

The interaction between Phe^229^ and Phe^279^ from IL-6R and the cytokine is mimicked by two hydrophobic residues from the CDR loops of 68F2 (Fig. 5, *B* and *C*, and electron density plot in supplemental Fig. 2). Here the hydrophobic side chain of Tyr^32^ (Kabat numbering 30) located in the CDR1 loop of the variable domain light chain superimposes on Phe^229^. Similarly, the side chain of Val^104^ (Kabat numbering 99) of the CDR3 loop of the heavy chain takes over the role of Phe^279^. Therefore, 68F2 can be seen as a true structural mimic of IL-6R: it occupies the same interaction site with and provides similar key interacting residues to anchor points on the IL-6 surface.

##### Side Chain of HCDR3 Residue Tryptophan of 61H7 Occupies a Cavity of Site I

The interaction surface of 61H7 on IL-6 only partly overlaps with that of IL-6R. Nevertheless, this overlap contains the cavity that accommodates hot spot residue Phe^229^ in the complex between IL-6 and IL-6R. Again, the position and interactions of Phe^229^ are mimicked by a hydrophobic residue contributed by the Fab fragment Trp^102^ (Kabat numbering 98) located in the CDR3 loop of the heavy chain (Fig. 5, *D* and *F*).

Together with our observations concerning 68F2, it seems that both antibodies converged to hit an epitope that is critical to block the interaction between IL-6 and IL-6R. Interestingly, the amount of non-polar surface buried upon the binding of 61H7 is almost identical to what is buried by IL-6R, the difference between 61H7 and IL-6R binding lying only in the amount of buried polar surface.

Mutation studies were performed to investigate the relevance of this HCDR3 residue in interaction with IL-6. These changes were made in the context of simultaneous mutation of methionine at position 104 (Kabat numbering 100) into alanine or leucine; this additional mutation was made to avoid potential oxidation leading to instability of the antibody. Periplasmic fractions containing Fabs were prepared from all mutants and were applied for off rate measurements using surface plasmon resonance (Table 3 and supplemental Fig. S3). For both versions with alanine or leucine on position 104 (100 in Kabat), the mutants containing phenylalanine on position 102 (98 in Kabat) had the best off rates, just a bit worse as compared with the parental one with tryptophan. The mutant with tyrosine was closely behind the phenylalanine containing version with leucine at position 104, whereas the similar mutant with alanine at position 104 also had one of the best off rates. In contrast, small hydrophobics such as Val or hydrophimics at position 102 led to an increase of up to two orders of magnitude in the off rate. This indicates a similar function as the hot spot residue of Trp^102^ for IL-6 recognition as is Phe^229^ in IL-6R.

**TABLE 3:** **Off rates (*k_d_* s^−1^) for 61H7 HCDR3 tryptophan 102 (Kabat numbering 98) mutants as measured on Biacore to assess the effect of mutating this key residue in the context of simultaneous mutation of methionine at position 104 (Kabat numbering 100) into leucine (Leu^104^) or alanine (Ala^104^)** See text for more details. ND, not determined.

Amino acid 102	Met^104^	Leu^104^	Ala^104^
Trp	4.7E-5	5.5E-5	8.3E-5
Phe	ND	2.2E-4	9.2E-5
Tyr	ND	3.1E-4	3.8E-4
Gln	ND	4.2E-4	ND
Met	ND	5.9E-4	7.4E-4
Val	ND	7.2E-4	1.2E-3
Cys	ND	7.7E-4	10.0E-4
Ser	ND	7.8E-4	ND
Leu	ND	8.4E-4	1.1E-3
Gly	ND	9.5E-4	1.8E-3
Arg	ND	1.0E-3	1.2E-3
Pro	ND	1.1E-3	ND
His	ND	ND	2.8E-4
Glu	ND	ND	7.4E-4
Asn	ND	ND	9.5E-4
Ile	ND	ND	9.6E-4
Lys	ND	ND	1.4E-3
Thr	ND	ND	1.6E-3
Ala	ND	ND	1.8E-3

## Discussion

In this paper, we present the crystal structures of two competing antibodies able to directly block the interaction between IL-6 and its receptor IL-6R. Of these, 68F2, the antibody with the highest (femtomolar) potency displays a very large surface (1156 Å^2^) of interaction with IL-6, whereas 61H7 has a smaller interaction surface (940 Å^2^). Although both antibodies 61H7 and 68F2 were obtained from random combinatorial Fab libraries, the light chains contribute significantly to target binding (for 61H7, 40% of the interaction surface is derived from VL, and for 68F2, 50% of the interaction surface is derived from VL). Light chains are usually less important in determining specificity for their antigen, whereas the VH domain typically dominates in a number of contacts (32). Serious doubts were raised concerning the identification of original heavy/light chain paired antibodies from combinatorial libraries, because target specific antibodies isolated from such libraries often show promiscuous pairing, *i.e.* the heavy chain determines antigen binding (for instance the anti-HIV antibody B12 (33) and the anti-influenza antibody CR6261 (34)); the light chain does not contribute significantly and can thus be replaced by other light chains.

Our antibodies were screened for their capability to compete against the binding of labeled IL-6 to coated recombinant IL-6R. Indeed, the structure of the two Fabs complexes reveals that 61H7 sterically hinders the interaction with IL-6R via its VL, whereas for 68F2 mainly the VH position overlaps with the binding of the receptor to IL-6. When zooming in on the epitope recognized by 61H7, we observed that the hydrophobic side chain of a HCDR3 residue occupies the site I cleft. The crystal structure of IL-6 in complex with its receptor and the signaling receptor gp130 (giving a hexameric complex) was determined by Boulanger *et al.* (30). IL-6 forms a non-signaling complex with IL-6R through interactions involving site I. Another site, site II is a composite epitope formed by the binary complex of IL-6 and IL-6R. Finally, interaction of site III with gp130 forms the signaling complex. The structure of the complex of IL-6 with its receptors revealed that the Phe^229^ side chain of IL-6R penetrates into the site I cavity. This amino acid was named the hot spot residue, because mutagenesis studies demonstrated its critical role in the cytokine/receptor interaction. Mutation of this residue to valine or serine completely abolishes IL-6R binding to IL-6 (28). Tryptophan residue 102 (98 in Kabat numbering), central in the heavy chain CDR3 loop of Fab 61H7, occupies the site I cavity. This suggests that it blocks the critical epitope in IL-6 and disrupts its interaction with IL-6R, hence mimicking the cytokine/receptor interaction (Fig. 5*E*). Accordingly, the human growth hormone receptor Trp^169^ side chain also occupies a similarly localized cavity on the surface of the human growth hormone that, like IL-6, is also a four-helix bundle cytokine (35).

The same kind of interaction mimicking mechanism was also found for Fab 68F2. Its light chain CDR1 Tyr^32^ side chain (Kabat number 30) also fills the IL-6 site I cleft, thereby mimicking the interaction of the earlier-mentioned hot spot IL-6R Phe^229^ residue. This strikingly illustrates the relevance of the light chain in the target binding of an antibody originating from a random combinatorial library. In addition the side chain of the HCDR3 residue Val^104^ (Kabat number 99) of 68F2 fills a complementary site of the site I crevice in a similar way as IL-6R Phe^279^. Next to Phe^229^, which accounts for 28% of the interaction surface with IL-6, the other phenylalanine (at position 279) accounts for 20% of the total interface, making it the second most important residue for interaction. This suggests that interactions of these two residues (Tyr^32^ of VL and Val^104^ of VH) with site I cavity makes 68F2 an extremely good antagonistic antibody and explains its femtomolar potency as measured in the sensitive bioassay.

The x-ray analysis reveals structural mimicry for the interactions of Fabs 61H7 and 68F2 with IL-6, as compared with the IL6/IL-6R interaction. In Fab 61H7 HCDR3 Trp^102^ side chain binds into the cavity of IL-6 site I, acting as a hot spot residue of IL-6R. In Fab 68F2 LCDR1 residue Tyr^32^ and HCDR3 residue Val^104^ play the same role. Such close structural and functional mimicry is rare. Mostly, antibodies that mimic ligand interactions on the antigen occupy a common antigen surface, but the atomic details of the interactions are usually very different. As an illustration, GC-1008, an antibody against human TGF-β that essentially recognizes the same epitope as the TGF-β receptors, is not able to distinguish between the three TGF-β variants because of a lack of specific interactions with two key specificity-determining arginine residues (36). The first occurrence of an antibody mimicking protein-ligand interactions was observed with the binding of a camelid single (heavy) chain antibody to lysozyme. In this complex the long CDR3 loop not only follows the path of the carbohydrate substrate of lysozyme but also establishes similar specific hydrogen bonds and hydrophobic contacts (37). Such cases, however, remain rare. To our knowledge, for conventional antibodies a unique case concerns the similarities between the recognition of the HIV-1 gp120 envelope glycoprotein by its CD4 receptor and by the neutralizing antibody VRC01 (38). In this case, however, the CD4 receptor domain interacting with gp120 has itself an immunoglobulin fold. It can thus be directly superimposed on the VH domain of VRC01, resulting in nearly identical interactions with the main chain atoms of the C″ strands and a common salt bridge via an arginine at the end of the D strand. Altogether, the data presented here reveal the structural basis of the extremely high affinity of two anti-IL-6 antibodies and illustrate that the diversity of antibodies following upon llama immunization is a good source of highly functional antibodies able to mimic key interactions of the IL6·IL-6R natural interface.

## Author Contributions

C. B., N. O., A. K.-R., A. S., and E. H. performed the experiments; A. D., S. S., amd C. C. determined the x-ray structure; C. B., N. D. J., A. H., and R. L. analyzed the data and contributed to the manuscript and figure preparation; and C. B., A. K., and H. d. H. conceived and coordinated the study. All authors reviewed the results and approved the final version of the manuscript.

## Supplementary Material

Supplemental Data

## Footnotes

3 The abbreviations used are:

IL-6R: IL-6 receptor
RMSD: root mean square deviation
d-PBS: Dulbecco's PBS
SPR: surface plasmon resonance
VH: variable heavy chain domain
VL: variable light chain domain.
