# Supplementary material for: Structural Mimicry of Receptor Interaction by Antagonistic Interleukin-6 (IL-6) Antibodies
Source: J Biol Chem. 2016 Apr 27;291(26):13846–54. doi: 10.1074/jbc.M115.695528 (PMC4919466; doi:10.1074/jbc.M115.695528)
Supplement: Supplemental Data [file supp_291_26_13846__index.html]

Structural mimicry of receptor interaction by antagonistic IL-6 antibodies — Structural Mimicry of Receptor Interaction by Antagonistic Interleukin-6 (IL-6) Antibodies — IL-6 Receptor Interaction Mimicked by Antibodies — Supplemental Data 

# Structural Mimicry of Receptor Interaction by Antagonistic Interleukin-6 (IL-6) Antibodies

## Supplemental Data

- Supplemental figure 1 (.pdf, 241 KB) - Supplemental figure 1
- Supplemental figure 2 (.pdf, 419 KB) - Supplemental figure 2
- Supplemental figure 3 (.pdf, 575 KB) - Supplemental figure 3
- Supplemental table S1 (.pdf, 18 KB) - Supplemental table S1
- Supplemental table S2 (.pdf, 97 KB) - Supplemental table S2
