# Supplementary material for: Structural Mimicry of Receptor Interaction by Antagonistic Interleukin-6 (IL-6) Antibodies
Source: J Biol Chem. 2016 Apr 27;291(26):13846–54. doi: 10.1074/jbc.M115.695528 (PMC4919466; doi:10.1074/jbc.M115.695528)
Supplement: Supplemental Data [file 10.1074_M115.695528_jbc.M115.695528-1.pdf]

Supplemental fig 1

Material and method:

Affinity measurements were performed on a Biacore T200 (GE Healthcare). Purified antibodies 61H7 and 68F2 were immobilized on separate channels on a Biacore CM5 chip using EDC-NHS coupling. The final immobilization level was approximately 500 RU of each.

Serial dilutions of recombinant hIL-6 (ImmunoTools, Germany) diluted in HBS-EP buffer (highest concentration 52.9 nM, 3-fold dilutions) were injected for 120 seconds at a flow rate of 30 µl/min, followed by a 30 min dissociation step. Between IL-6 injections, full surface regeneration was achieved by injection of 10mM glycine pH 1.5.

Curves were fitted according to a 1:1 binding model using the T200 Evaluation software. For 61H7, the kinetic constant kd was outside the limits that can be measured by the instrument, whereas for 68F2 this was approaching the limits of the instrument.

Legend

Supplementary Fig 1: Binding and dissociation curves of hIL-6 to the coated anti-IL-6 antibodies. Serial dilutions of IL-6 were injected on immobilized 61H7 (A) or 68F2 (B) for 120 seconds, followed by a 30 min. dissociation wash. Curves were fitted according to a 1:1 binding model and fittings (black lines) were superimposed on the binding data (coloured curves).

Supplementary Fig. 1A: mAb 61H7

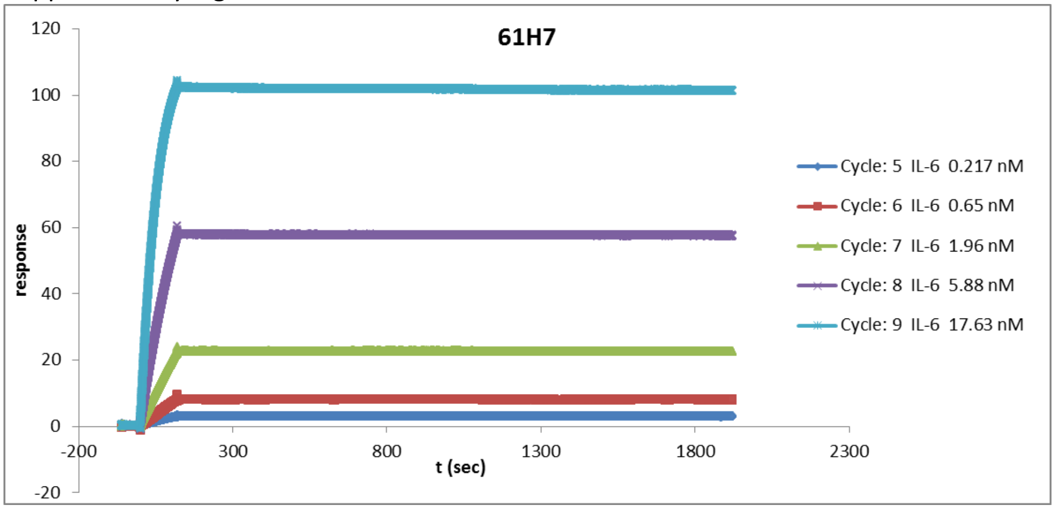

Supplementary Fig. 1B: mAb 68F2

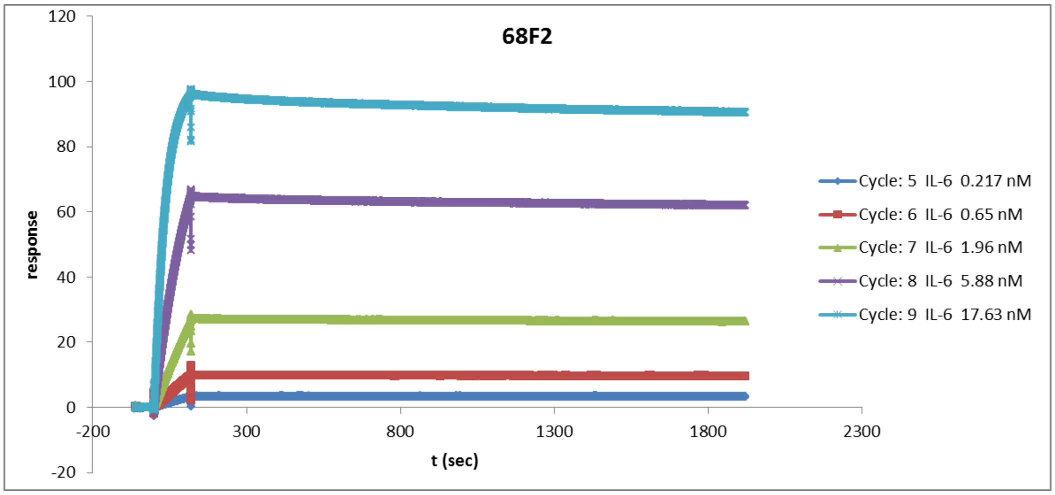

Overview of the kinetic fitting #1, according to a 1:1 binding model.

	ka (1/Ms)	kd (1/s)	KD (M)	Rmax (RU)	tc	Chi² (RU²)	U-value
61H7	1.50E+06	4.91E-06	3.27E-12	109.9	8.83E+07	0.0159	12
68F2	2.08E+06	2.77E-05	1.34E-11	96.77	1.15E+08	0.0532	1

Overview of the kinetic fitting #2, according to a 1:1 binding model.

	ka (1/Ms)	kd (1/s)	KD (M)	Rmax (RU)	tc	Chi² (RU²)	U-value
61H7	9.46E+05	5.97E-06	6.31E-12	118.7	1.52E+09	0.203	12
68F2	1.35E+06	2.87E-05	2.13E-11	103.1	1.89E+19	1.51	5
