# Supplementary material for: Structural Mimicry of Receptor Interaction by Antagonistic Interleukin-6 (IL-6) Antibodies
Source: J Biol Chem. 2016 Apr 27;291(26):13846–54. doi: 10.1074/jbc.M115.695528 (PMC4919466; doi:10.1074/jbc.M115.695528)

Supplemental figure 2

The electron density of key regions of interaction between IL6 and the antibodies 61H7 (A, W102 in heavy chain CDR3) and 68F2 (B, Y32 in light chain CDR1 and C, V104 in heavy chain CDR3).

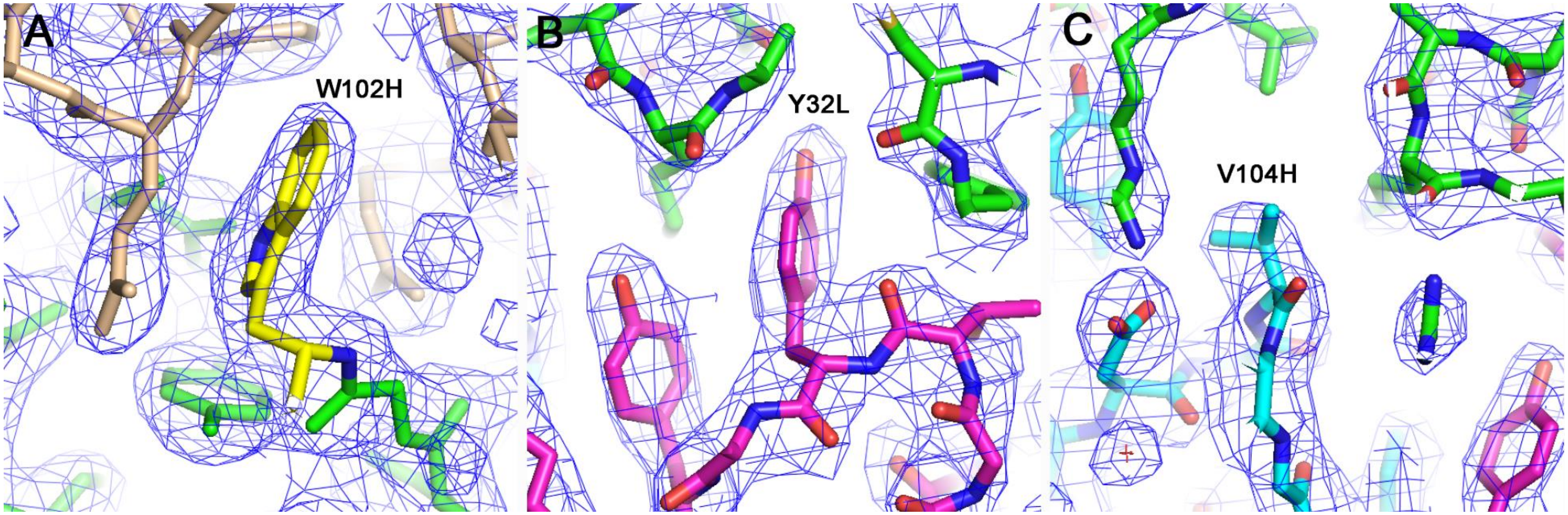

Supplement: Supplemental Data [file 10.1074_M115.695528_jbc.M115.695528-2.pdf]
