# Supplementary material for: Structural Mimicry of Receptor Interaction by Antagonistic Interleukin-6 (IL-6) Antibodies
Source: J Biol Chem. 2016 Apr 27;291(26):13846–54. doi: 10.1074/jbc.M115.695528 (PMC4919466; doi:10.1074/jbc.M115.695528)
Supplement: Supplemental Data [file 10.1074_M115.695528_jbc.M115.695528-3.pdf]

Supplemental figure 3

Method.

The tryptophan at position 102 (Kabat numbering 98) of the antibody 61H7 was randomized using degenerated primers. After cloning into a bacterial expression vector and induction of production in the bacterial periplasm (as previously described (22)), the Fab extracted from the periplasm were tested for binding to IL6 by SPR using a Biacore 3000 and low IL-6 coating (~75-100 RU) on a CM5-Chip (GE Healthcare) to determine the k_{off} of each Fab produced in periplasmic fractions. k_{off} was measured over a10 minute washing period (30 µl/min) and evaluated using the BIAevaluation software. After sequencing, the off rate of the clones with the same amino acid was combined and averaged (Table below and Table 3 in article).

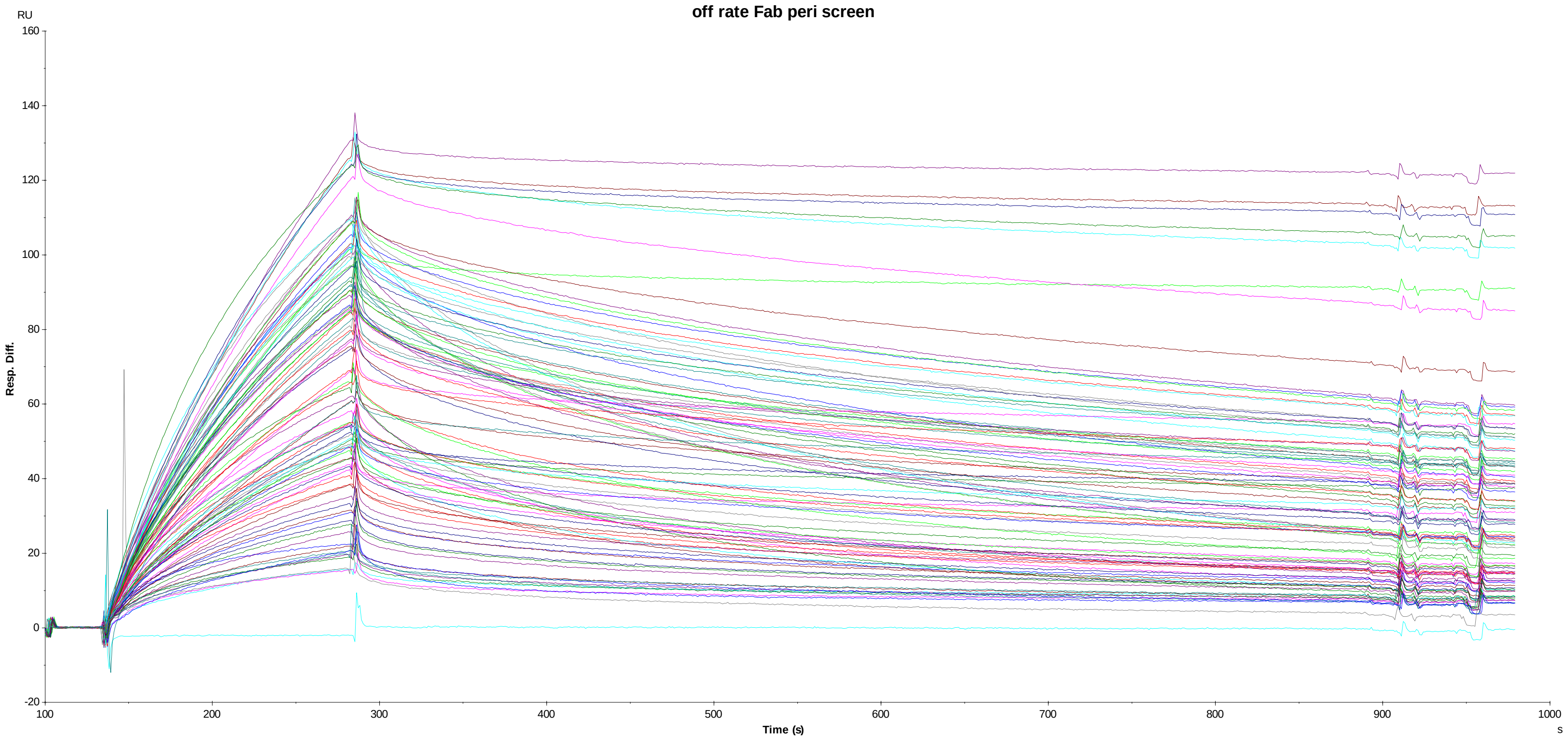

Analysis:

M100A/L	W98X	kd (s-1)	n
M	WGM	4,67E-05	n=3
L	WGL	5,50E-05	n=1
L	FGL	2,20E-04	n=1
L	YGL	3,10E-04	n=1
L	QGL	4,20E-04	n=1
L	MGL	5,90E-04	n=1
L	VGL	7,17E-04	n=3
L	CGL	7,70E-04	n=2
L	SGL	7,77E-04	n=5
L	LGL	8,35E-04	n=6
L	GGL	9,46E-04	n=7
L	RGL	1,00E-03	n=2
L	PGL	1,10E-03	n=2

M100A/L	W98X	kd (s-1)	n
M	WGM	4,67E-05	n=3
A	WGA	8,30E-05	n=1
A	FGA	9,20E-05	n=1
A	HGA	2,83E-04	n=3
A	YGA	3,75E-04	n=2
A	MGA	7,35E-04	n=2
A	EGA	7,45E-04	n=2
A	NGA	9,50E-04	n=1
A	IGA	9,63E-04	n=3
A	CGA	9,95E-04	n=2
A	LGA	1,05E-03	n=5
A	*GA	1,05E-03	n=2
A	VGA	1,23E-03	n=4
A	RGA	1,25E-03	n=5
A	KGA	1,45E-03	n=2
A	TGA	1,55E-03	n=2
A	GGA	1,77E-03	n=6
A	AGA	1,85E-03	n=2
