# Supplementary material for: Structural Mimicry of Receptor Interaction by Antagonistic Interleukin-6 (IL-6) Antibodies
Source: J Biol Chem. 2016 Apr 27;291(26):13846–54. doi: 10.1074/jbc.M115.695528 (PMC4919466; doi:10.1074/jbc.M115.695528)
Supplement: Supplemental Data [file 10.1074_M115.695528_jbc.M115.695528-4.pdf]

Supplementary Table S1. Hydrogen bonds and salt bridges in 61H7:IL-6 complex.

		61H7 residue structure	Kabat numbering	Distance (Å)	IL-6 residue
**Hydrogen bonds**	Light chain-CDR1	THR 30[OG1]	**THR 28**	3.21	LYS 27[NZ]
		SER 32[OG]	**SER 30**	2.66	ASP 26[OD1]
		ASN 33[ND2]	**ASN 31**	2.99	GLU 23[OE2]
		TYR 34[OH]	**TYR 32**	2.62	ARG 30[NH1]
		TYR 34[OH]	**TYR 32**	2.55	ASP 26[OD2]
	Light chain-CDR3	ASP 93[OD2]	**ASP 91**	2.72	ARG 182[NH1]
		ASP 93[OD2]	**ASP 91**	2.99	ARG 182[NH2]
		GLY 95[O]	**GLY 93**	3.45	SER 22[N]
		GLY 95[N]	**GLY 93**	2.91	GLU 23[OE1]
		GLY 95[O]	**GLY 93**	3.06	GLU 23[N]
		ASP 96[OD1]	**ASP 94**	3.04	SER 22[OG]
		ASP 96[OD1]	**ASP 94**	2.94	SER 22[N]
	Heavy chain-CDR1	THR 28[OG1]	**THR 28**	3.44	GLN 75[NE2]
		SER 31[OG]	**SER 31**	2.96	GLN 75[O]
		ARG 33[N]	**ARG 33**	2.73	GLN 183[OE1]
	Heavy chain-CDR2	ALA 53[N]	**ALA 52a**	2.76	GLN 183[O]
		ALA 53[N]	**ALA 52a**	3.66	GLU 80[OE1]
		GLY 54[N]	**GLY 53**	3.23	GLU 80[OE1]
		GLY 56[N]	**GLY 55**	2.78	GLU 80[OE2]
		TYR 59[OH]	**TYR 58**	3.54	MET 184[O]
	Heavy chain-CDR3	ARG 99[NH1]	**ARG 95**	2.71	ARG 182[O]
		ALA 100[O]	**ALA 96**	2.94	GLN 183[NE2]
**Salt bridges**	Light chain-CDR3	ASP 93[OD2]	**ASP 91**	2.72	ARG 182[NH1]
		ASP 93[OD2]	**ASP 91**	2.99	ARG 182[NH2]
