# Supplementary material for: Structural Mimicry of Receptor Interaction by Antagonistic Interleukin-6 (IL-6) Antibodies
Source: J Biol Chem. 2016 Apr 27;291(26):13846–54. doi: 10.1074/jbc.M115.695528 (PMC4919466; doi:10.1074/jbc.M115.695528)
Supplement: Supplemental Data [file 10.1074_M115.695528_jbc.M115.695528-5.pdf]

Supplementary Table S2. Hydrogen bonds and salt bridges in 68F2:IL-6 complex.

		**68F2 residue structure**	**Kabat numbering**	**Distance (Å)**	**IL-6 residue**
**Hydrogen bonds**	Light chain- CDR1	ASN 26[O]	**ASN 27**	3.60	SER 76[OG]
		TYR 32[OH]	**TYR 30**	3.43	SER 176[O]
		THR 31[N]	**THR 29**	3.55	SER 76[OG]
		THR 31[O]	**THR 29**	3.42	GLN 75[N]
	Light chain- CDR3	ASN 95[ND2]	**ASN 93**	3.77	MET 67[O]
		ASN 97[OD1]	**ASN 95**	3.73	ARG 179[NH2]
	Heavy chain- CDR1	TYR 33[OH]	**TYR 33**	3.47	ARG 30[O]
		TYR 33[OH]	**TYR 33**	2.16	ASP 34[OD1]
		ARG 32[NE]	**ARG 32**	3.20	ASP 34[OD2]
		ARG 32[NH2]	**ARG 32**	3.28	ASP 34[OD2]
	Heavy chain- CDR2	TYR 60[OH]	**TYR 58**	2.59	GLU 172[OE2]
		ASP 54[OD2]	**ASP 52**	3.37	LYS 171[NZ]
		ASP 56[OD1]	**ASP 54**	2.90	SER 37[OG]
		ASP 58[OD1]	**ASP 56**	3.86	HIS 164[NE2]
		ASP 58[OD2]	**ASP 56**	3.42	LYS 171[NZ]
		THR 59[O]	**THR 57**	2.52	ARG 168[NH2]
		TYR 60[OH]	**TYR 58**	3.69	ARG 168[NE]
		TYR 60[OH]	**TYR 58**	3.15	LYS 171[NZ]
	Heavy chain- CDR3	ASP 102[OD2]	**ASP 97**	2.77	ARG 30[NH2]
		VAL 104[O]	**VAL 99**	3.69	ARG 179[NH1]
**Salt bridges**	Heavy chain- CDR1	ARG 32[NE]	**ARG 30**	3.20	ASP 34[OD2]
		ARG 32[NH2]	**ARG 30**	3.28	ASP 34[OD2]
	Heavy chain- CDR2	ASP 54[OD2]	**ASP 52**	3.37	LYS 171[NZ]
		ASP 58[OD1]	**ASP 56**	3.86	HIS 164[NE2]
		ASP 58[OD2]	**ASP 56**	3.42	LYS 171[NZ]
	Heavy chain- CDR3	ASP 102[OD1]	**ASP 97**	3.40	ARG 30[NH2]
		ASP 102[OD2]	**ASP 97**	2.77	ARG 30[NH2]
